# Hydrologic variation influences stream fish assemblage dynamics through flow regime and drought

**DOI:** 10.1038/s41598-021-89632-3

**Published:** 2021-05-21

**Authors:** Daniel D. Magoulick, Matthew P. Dekar, Shawn W. Hodges, Mandy K. Scott, Michael R. Rabalais, Christopher M. Bare

**Affiliations:** 1 U.S. Geological Survey, Arkansas Cooperative Fish and Wildlife Research Unit, Department of Biological Sciences, University of Arkansas, Fayetteville, AR 72701 USA; 2 Arkansas Cooperative Fish and Wildlife Research Unit, Department of Biological Sciences, University of Arkansas, Fayetteville, AR 72701 USA

**Keywords:** Community ecology, Freshwater ecology

## Abstract

Hydrologic variation can play a major role in structuring stream fish assemblages and relationships between hydrology and biology are likely to be influenced by flow regime. We hypothesized that more variable flow regimes would have lower and more variable species richness, higher species turnover and lower assemblage stability, and greater abiotic environment-fish relationships than more stable flow regimes. We sampled habitats (pool, run, and riffle) in three Runoff/Intermittent Flashy streams (highly variable flow regime) and three Groundwater Flashy streams (less variable flow regime) seasonally (spring, early summer, summer and autumn) in 2002 (drought year) and 2003 (wet year). We used backpack electrofishing and three-pass removal techniques to estimate fish species richness, abundance and density. Fish species richness and abundance remained relatively stable within streams and across seasons, but densities changed substantially as a result of decreased habitat volume. Mixed model analysis showed weak response variable-habitat relationships with strong season effects in 2002, and stronger habitat relationships and no season effect in 2003, and flow regime was not important in structuring these relationships. Seasonal fish species turnover was significantly greater in 2002 than 2003, but did not differ between flow regimes. Fish assemblage stability was significantly lower in Runoff/Intermittent Flashy than Groundwater Flashy streams in 2002, but did not differ between flow regimes in 2003. Redundancy analysis showed fish species densities were well separated by flow regime in both years. Periodic and opportunistic species were characteristic of Runoff/Intermittent Flashy streams, whereas mainly equilibrium species were characteristic of Groundwater Flashy streams. We found that spatial and temporal variation in hydrology had a strong influence on fish assemblage dynamics in Ozark streams with lower assemblage stability and greater fluctuations in density in more hydrologically variable streams and years. Understanding relationships between fish assemblage structure and hydrologic variation is vital for conservation of fish biodiversity. Future work should consider addressing how alteration of hydrologic variation will affect biotic assemblages.

## Introduction

Hydrologic (flow) regimes can act as an overriding factor in shaping physical and biological components of stream systems^1,2^. Hydrologic variation is often a dominant factor structuring many stream communities^1,3^, potentially overriding other important abiotic and biotic factors^4^. In particular, stream fish assemblages are often associated with flow regimes and hydrologic variation^5,6^.

Natural hydrologic variation in streams within a region is typically related to climate, geology, watershed size, groundwater inputs and many other factors. These underlying factors responsible for hydrologic variation make it possible to classify streams into natural flow regimes that often cluster geographically and can be used as a basis for examining relationships between hydrology and biology^7^.

Poff et al.^7^ suggested that biology-hydrology relationships should be examined within flow regimes, because differing flow regimes even within the same region are likely to support distinct stream ecosystem structure and function^8^ which may mask biology-hydrology relationships. Determining relationships between fish assemblage structure and environmental variables in reference streams in various natural flow regimes can be an important step in conserving and managing stream systems. In addition, recognition of both biological and hydrological variation at multiple spatial scales is important to conserving natural flow-ecology relationships^9,10^.

Flow regimes and hydrologic variation may differ in effects on biota across ecoregions due to historical artifacts and high endemism. However, biological response to hydrologic variation should reflect predominant life history strategies and species traits^5,11^. Therefore, a trait-based approach may be valuable in examining response to hydrologic variation. Winemiller and Rose^12^ classified fishes into three major life history categories: opportunistic, periodic and equilibrium. Studies suggest that these life history categories should be related to hydrologic variability, with opportunistic strategy favored in high hydrologic variability systems^11,13^.

Previous research has examined the influence of hydrologic variation on fish assemblage structure^5,14–18^. However, relatively few studies have examined the influence of hydrologic variation on fish species turnover defined as the proportions of species that differ between time points. Taylor and Warren^19^ found that hydrologic variation was related to fish assemblage nestedness due to high extinction rates in areas with greater hydrologic variation. Fish beta diversity was greater in tributaries versus mainstem sites than between mainstem sites suggesting that species turnover may be greater due to hydrologic variation^20^. Conversely, Cook et al.^21^ found that annual fish species turnover did not explain patterns of nestedness in Virginia streams.

Headwater streams of the Ozark Plateau of Arkansas, USA show distinct flow regimes. Groundwater streams tend to be more stable, with less extreme seasonal drying, whereas runoff and intermittent streams tend to be flashy and experience frequent and intense drying during summer and autumn^22^. Leasure et al.^22^ classified streams of the Interior Highlands into seven natural flow regimes: Groundwater Stable, Groundwater, Groundwater Flashy, Perennial Runoff, Runoff Flashy, Intermittent Runoff and Intermittent Flashy. Many of the larger headwater streams in the Ozark Plateau are Groundwater Flashy and Runoff Flashy, with smaller headwater streams typically Intermittent Flashy in both ecoregions.

We examined fish assemblage structure in Ozark Plateau streams to determine temporal variation in fish species composition, relative abundance and its relationship to environmental variables in natural flow regimes. We hypothesized that the more variable flow regimes (Runoff Flashy and Intermittent Flashy) would have lower and more variable species richness, higher species turnover and lower assemblage stability, and greater abiotic environment-fish relationships than the more stable flow regime (Groundwater Flashy). We hypothesized that fish assemblages would be more temporally variable in Runoff Flashy/Intermittent Flashy than Groundwater Flashy streams due to greater hydrologic variability in these systems. We also expected that the more stable Groundwater Flashy streams would have more equilibrium species and the Runoff Flashy/Intermittent Flashy streams that experience strong seasonal flooding and drying would have more opportunistic and periodic species.

## Study area

**Figure 1:**
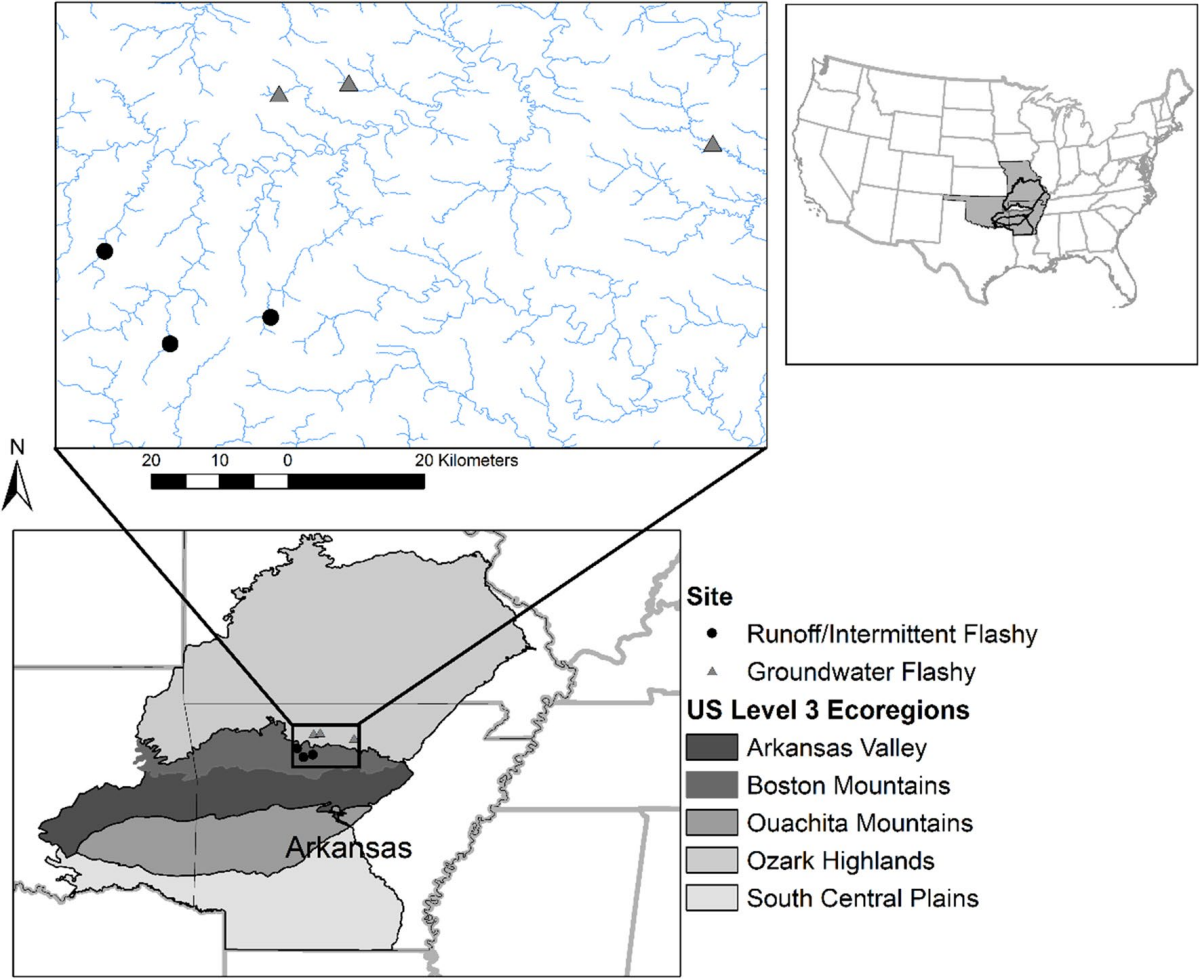
Map of study sites in Arkansas showing Runoff/Intermittent Flashy and Groundwater Flashy streams. Ecoregion boundaries also are displayed. Base map is GCS WGS84. Figure produced with ArcGIS 10.5.1 [https://www.esri.com/en-us/arcgis/about-arcgis/overview].

Study streams were in the Ozark Plateau, USA that is composed of the Ozark Highlands and the Boston Mountains (Fig. 1). In this region, stream substrate ranges from bedrock-boulder to gravel, and riffle-pool geomorphology is typical^23^. Land use in the Ozark Plateau is primarily forest dominated by oak and hickory and agriculture dominated by pasture and hay fields^23^. The Ozark Highlands has less elevation change^24,25^ and more nutrient-rich soils than the Boston Mountains contributing to higher percentage agricultural land in this region. Karst topography in the Ozark Highlands results in streams that are heavily spring-influenced, making them cooler with more stable flow regimes than Boston Mountain streams^22,25,26^.

We selected six headwater stream reaches: Bear (35° 46.1601′ N, 92° 48.0548′ W, second order, 34 sq km drainage), Cave (35° 51.4023′ N, 93° 1.1812′ W, second order, 32 sq km drainage), and Falling Water Creeks (35° 44.054′ N, 92° 56.014′ W, second order, 37 sq km drainage; Falling) in the runoff/intermittent flow regime, and Tomahawk (36° 3.888′ N, 92° 47.403′ W, third order, 20 sq km drainage), Water (36° 4.7519′ N, 92° 41.8947′ W, third order, 20 sq km drainage), and North Sylamore Creeks (35° 59.9641′ N, 92° 13.1154’W, fourth order, 120 sq km drainage, Sylamore) in the groundwater flow regime (Fig. 1). Streams were selected according to the following criteria: (1) wadeable for sampling, (2) similar stream width and depth, (3) sites had an intact riparian zone, and (4) sites were not affected by urban areas or dams. Reaches within the streams were selected on the basis of stream width and accessibility. Groundwater streams had significantly higher conductivity than runoff/intermittent streams, indicating the influence of groundwater and the underlying limestone and dolomite in these streams. All sites were within the White River drainage basin. Although average annual rainfall in the area ranges 1.0–1.2 m year^−1^^27^, reduced flow is characteristic from June through November.

Leasure et al.^22^ classified natural flow regimes in the Interior Highlands and the streams in the present study were predicted to be Intermittent Flashy (Cave and Tomahawk), Runoff Flashy (Bear and Falling) and Groundwater Flashy (Water and Sylamore). Groundwater Flashy streams never dried completely and had less daily flow variability and frequency of low flow spells, and greater base flow index than runoff-dominated streams. Conversely, Runoff Flashy and Intermittent Flashy streams had greater daily flow variability and frequency of low flow spells, and much lower base flow index than Groundwater Flashy streams, as well as experiencing 2–15 days (Runoff Flashy) or 1–3 months (Intermittent Flashy) of drying per year. Drying in these systems is often complete but can also lead to residual pools with some standing water. Ground-truthing suggested that all of these classifications were appropriate with the exception of the Tomahawk Creek site, which appeared to be Groundwater Flashy based on abiotic and biotic variables. Unlike Cave Creek, in which some habitat units dried completely during summer and autumn 2002 and autumn 2003, no habitat units in Tomahawk Creek dried completely during 2002 or 2003. Given this and given that the Tomahawk Creek sampled stream segment is just upstream of a stream segment classified as Groundwater Flashy^22^, we reclassified this stream segment as Groundwater Flashy for the present study.

## Methods

### Fish assemblage structure

We sampled each of the six sites on four occasions (spring, early summer, summer, autumn) in both 2002 and 2003 (so 6 * 4 * 2 years of sampling events minus the first two North Sylamore samples). Each of these 46 sampling events was conducted during a single day (Table 1). Stream reaches ranged 170–250 m long and encompassed multiple habitat units (pool, run, and riffle). Habitat units ranges 5–8 per stream reach. We determined habitat types by qualitatively assessing depth and flow rate of the stream. Riffles were relatively fast flowing, shallower water with noticeable surface aeration. Runs consisted of fast to moderately flowing water with unbroken surface flow. Slow moving, deeper water was pool habitat. Prior to sampling, block nets were placed at the upstream and downstream ends of the entire stream reach to be sampled to prevent the potential immigration or emigration of fish within the reach. Additionally, each habitat unit was closed to fish escape just prior to sampling by placement of block nets across the downstream and upstream boundaries of the habitat unit. One person operated the backpack electrofisher along with a two- or three-person team of netters while all moved upstream through the habitat and removed fish they encountered. A second and third pass were made within each habitat unit with an equivalent effort. Fish collected from each pass were held in separate containers, identified to species, and measured (total length, TL) to the nearest 10 mm. All fish were then returned to the habitat unit from which they were collected and the process was repeated at the next adjacent upstream habitat unit. This study followed relevant guidelines and regulations. All the experimental protocols were approved by University of Arkansas Institutional Animal Care and Use Committee protocol #01027. Reporting in this manuscript follows the recommendations in the ARRIVE guidelines^28^.

**Table 1:** Collection dates for seasonal fish sampling in Runoff/Intermittent Flashy (RIF) and Groundwater Flashy (GF) streams in 2002 and 2003.

Site	Flow regime	Date sampled			
		Spring	Early summer	Summer	Autumn
Bear	RIF	21-Apr-02	17-Jun-02	30-Jul-02	7-Oct-02
		14-Apr-03	10-Jun-03	13-Aug-03	28-Sep-03
Cave	RIF	15-Apr-02	11-Jun-02	1-Aug-02	8-Oct-02
		9-Apr-03	11-Jun-03	12-Aug-03	5-Oct-03
Falling Water	RIF	13-Apr-02	10-Jun-02	31-Jul-02	1-Oct-02
		8-Apr-03	9-Jun-03	28-Jul-03	27-Sep-03
N. Sylamore	GF			17-Jul-02	6-Oct-02
		11-Apr-03	12-Jun-03	29-Jul-03	26-Sep-03
Tomahawk	GF	14-Apr-02	4-Jun-02	2-Aug-02	5-Oct-02
		13-Apr-03	5-Jun-03	14-Aug-03	2-Oct-03
Water	GF	20-Apr-02	6-Jun-02	29-Jul-02	3-Oct-02
		10-Apr-03	4-Jun-03	11-Aug-03	4-Oct-03

### Physical–chemical environmental data collection

Habitat variables were measured in each habitat unit following electrofishing. Multiple cross-stream transects were established in each habitat, with the number of transects based on habitat unit length. The criteria for determining the number of transects in each habitat unit were, (1) a minimum of three transects for habitats ≤ 15 m long, (2) every 5 m for habitats > 15 m long and ≤ 50 m, and (3) a minimum of 10 transects in habitat units > 50 m long. Total depth, substrate size and mean current velocity were measured at three equidistant points along each cross-stream transect. Habitat length, maximum depth, and wetted widths at transects were measured in each habitat unit. Habitat unit area was calculated as length times mean width. Habitat unit volume was calculated as area times mean depth. Habitat units that dried completely were noted. We also examined habitat volume in each stream as an index of stream discharge and drying. Hydrologic variation was examined at the two streams with USGS gages, Bear Creek and North Sylamore Creek (gages were downstream of sampled sites), and we assumed that hydrologic variation was similar among other ungaged study streams.

### Data analysis

We used fish removal data from the three electrofishing pass samples in each year and season to produce population abundance estimates for each habitat unit for each species. We used the Zippin^29^ removal estimator via maximum-likelihood estimation in program MicroFish 3.0^30^. Estimated abundance of each species was then divided by the habitat unit area to derive species densities. For stream site estimates of abundance or density we summed the habitat unit estimates at a given site by season by year.

To determine how differences in capture probability by season would affect population estimates we ran simulations in the program MARK using the CAPTURE module. We used the Zippin removal estimator with constant capture probability over three sampling occasions (i.e., three pass removal sampling) and ran 1000 simulations for each combination of capture probability and population size. We varied capture probability from 0.10 to 0.90 and population size from 50 to 300 individuals.

Ordination was used to determine relationships between fish species densities and composition by flow regime and season. Redundancy analysis (RDA) was used to ordinate streams by seasons based on fish species relative densities in CANOCO 4.5^31^. Stream and season were used as explanatory variables and covariables^31^. We focused on streams for this analysis to examine fish species composition among streams, but we also examined influence of flow regime on fish assemblage structure. A linear model and RDA were appropriate because a preliminary detrended canonical correspondence analysis showed that gradient lengths were < 3 standard deviations^32^. Partial RDAs and Monte Carlo permutation tests^31^ were used to test the null hypothesis that season and stream categories had no relationship to fish species densities.

We used three-way ANOVAs to examine the effect of flow regime, season and year on total fish density, total abundance, fish species richness and habitat volume. For the univariate analyses we were focused on habitat volume and drying, so for these analyses density was calculated as number of fish m^−3^. When significant interactions were found, we used one-way ANOVA and Tukey’s tests to examine differences among seasons separately for each flow regime.

We used mixed models to examine relationships between response variables species richness, total abundance and total density (number m^−2^) and fixed effects substrate size, habitat volume and current velocity, as well as season and flow regime. Habitat unit was nested in stream and treated as random variables. A separate mixed model analysis was done for each year. Each model contained fixed effects season and flow regime and all additive combinations of depth, substrate and current velocity were used in the candidate models, so six models per year were examined. Continuous predictor variables were standardized. Akaike Information Criterion corrected for small sample size (AICc) was used to select best models. Models within two AICc scores were considered equally plausible^33^. For this analysis packages “lme4” and “AICcmodavg” were used in R^34^.

Seasonal species turnover rates and assemblage stability were calculated for each year using the package “codyn”^35^ in R^34^. Species turnover is the proportion of species that differ between time points and assemblage stability is the temporal mean divided by the temporal standard deviation. Species turnover was compared among flow regime, season and year with three-way ANOVA. Effect of flow regime and year on assemblage stability were examined with two-way ANOVA. For all linear models, residuals indicated that total fish density, total abundance, habitat volume and assemblage stability required log_10_ transformation to meet homogeneity of variance assumptions, whereas species richness and species turnover required no transformation. ANOVAs were conducted in SYSTAT 13 (Systat Software, San Jose, CA).

We assigned species to life-history strategies (Periodic, Opportunistic, Equilibrium) based on Robison and Buchanan^36^, Pflieger^37^, Winemiller^38^ and Hoeinghaus et al.^39^. For species that were between categories, we used the approach of Hoeinghaus et al.^39^ and provided combined categories (e.g., Opportunistic/Equilibrium). We determined characteristic species for ecoregion/flow regimes based on RDA and species relative densities. We defined characteristic species as those that were abundant in a particular flow regime.

## Results

### Hydrologic variation

**Figure 2:**
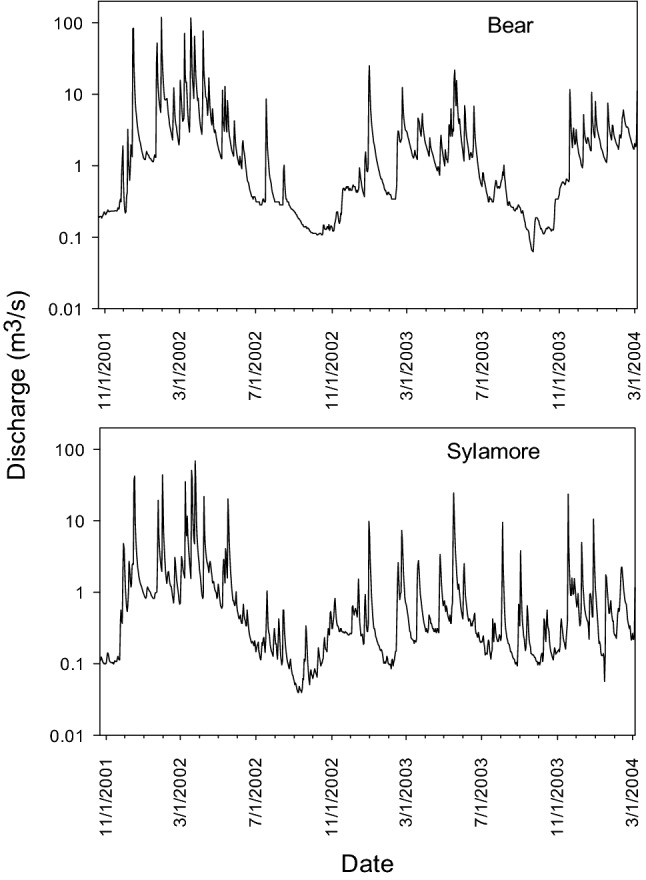
Bear (Runoff/Intermittent Flashy) and Sylamore (Groundwater Flashy) Creek hydrographs showing discharge (m^3^ s^−1^) from 2002 to 2003. Source data USGS NWIS. Figure produced with R^34^.

**Figure 3:**
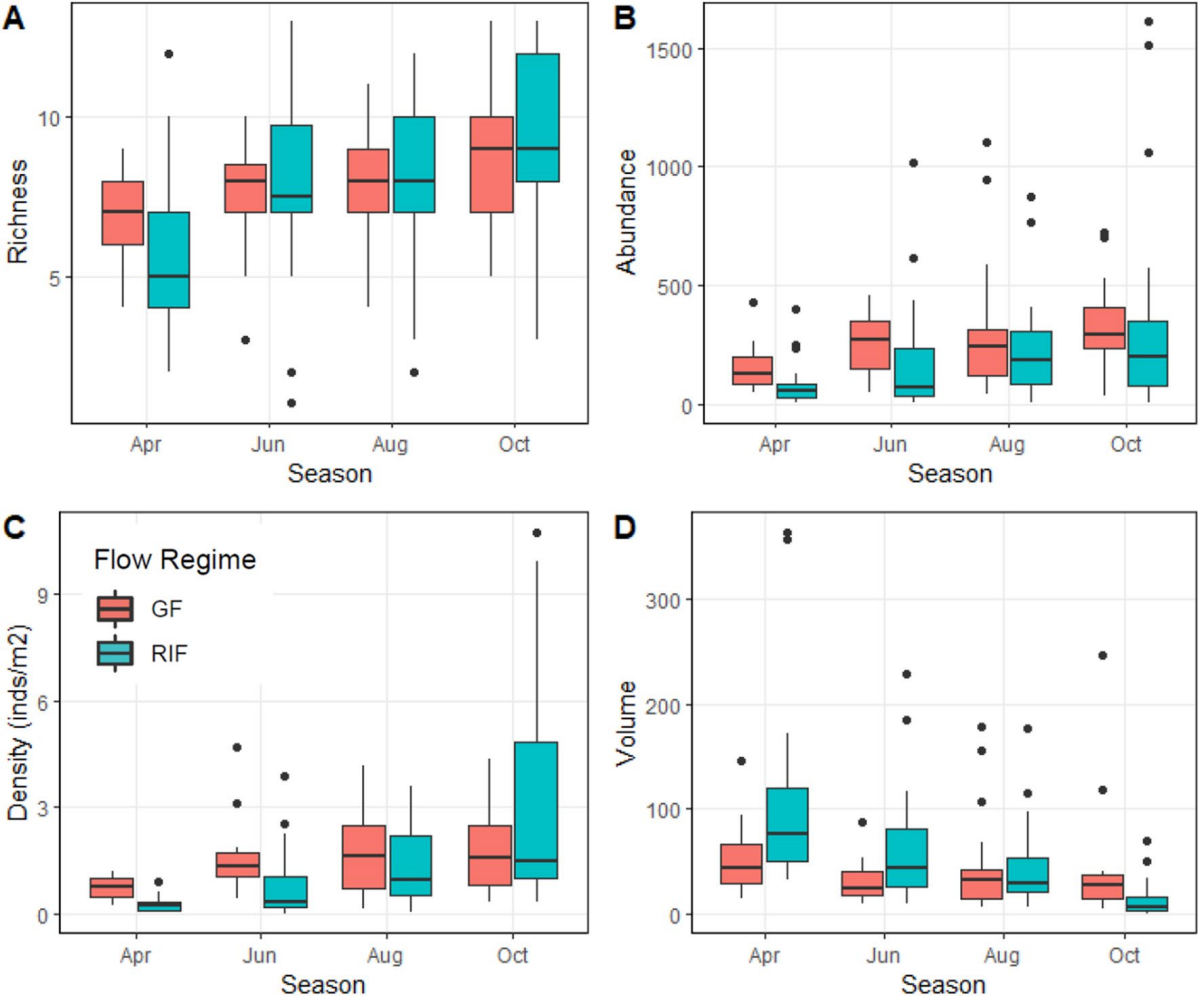
Boxplots of fish species richness (**A**), fish total abundance (**B**), fish total density (**C**) and (**D**) habitat volume (m^3^) in Runoff/Intermittent Flashy (RIF) and Groundwater Flashy (GF) streams in 2002. Box indicates median and interquartile range, whiskers show range, and dots indicates outliers (1.5 × interquartile range). Figure produced with R^34^.

**Figure 4:**
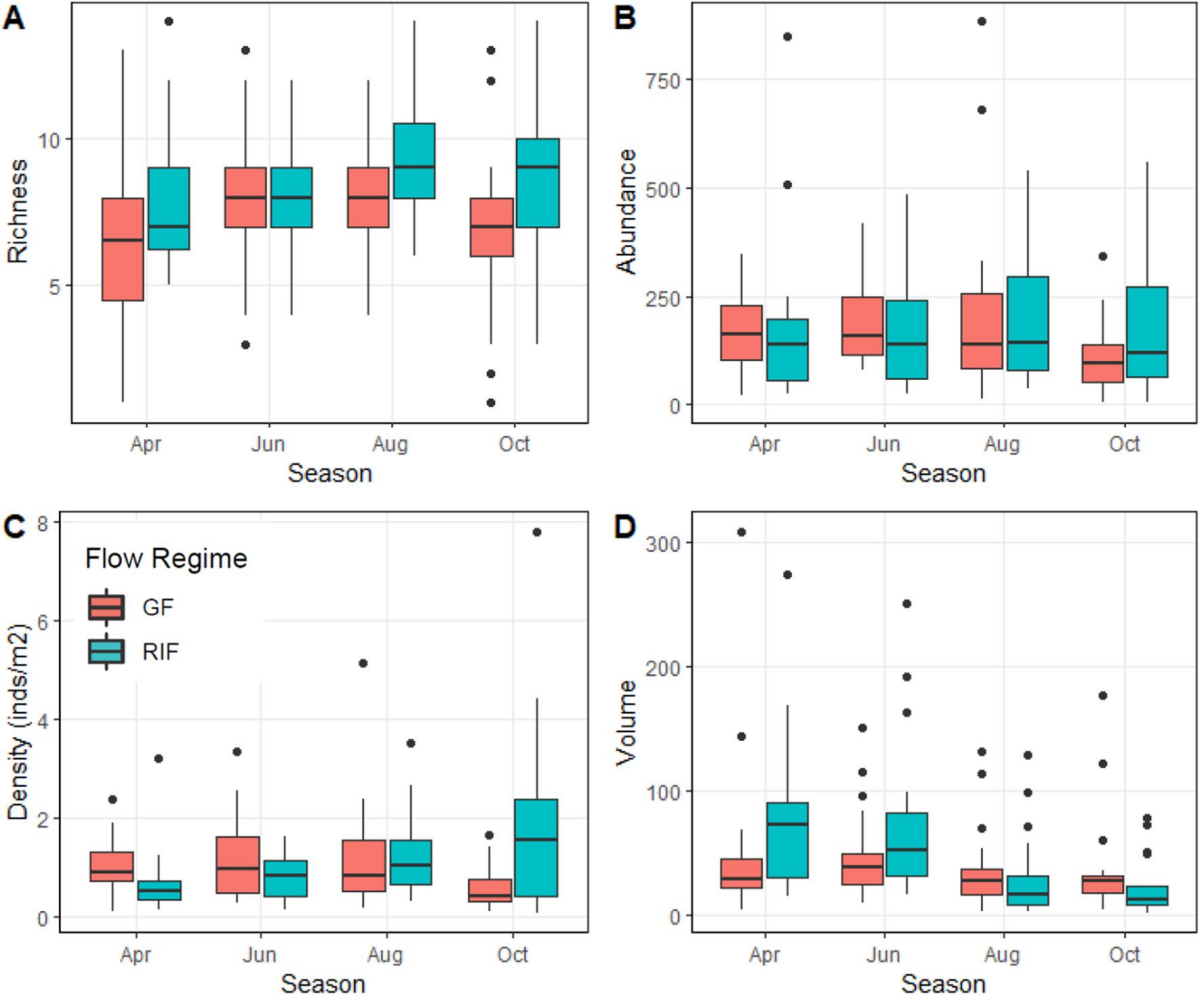
Boxplots of fish species richness (**A**), fish total abundance (**B**), fish total density (**C**) and (**D**) habitat volume (m^3^) in Runoff/Intermittent Flashy (RIF) and Groundwater Flashy (GF) streams in 2003. Boxplots are as in Fig. 3. Figure produced with R^34^.

Peak flows were greater in Bear Creek than Sylamore Creek despite a greater drainage area in Sylamore Creek, giving Bear Creek over twice the unit peak discharge as Sylamore Creek (Fig. 2). Drying occurred in both Bear and Sylamore Creeks in 2002, but happened only in Bear Creek in 2003. Hydrologic variation was greater in 2002 than 2003, with greater magnitude and frequency of high flows in 2002 than 2003 (Fig. 2). Drying was extreme in both years in Runoff/Intermittent Flashy streams, but variation between high and low flows and resulting habitat volume was greater in 2002 than 2003 (Figs. 3, 4). Some habitat units dried completely in Runoff/Intermittent Flashy streams in 2002 and 2003. Drying was extreme in 2002 in Groundwater Flashy streams, but mainly occurred between spring and early summer, whereas in 2003 Groundwater Flashy streams had much more stable flow (Figs. 3, 4). No habitat units dried completely in either year in any Groundwater Flashy stream.

There was no significant flow regime by season by year interaction for habitat volume (ANOVA *p* = 0.687, Figs. 3, 4). However, there was a significant flow regime by season interaction (ANOVA *p* = 0.011, Figs. 3, 4). In Runoff/Intermittent Flashy streams, habitat volume was significantly less in autumn than other seasons (Tukey’s *p* < 0.001) and less in summer than spring (Tukey’s *p* = 0.018), whereas Groundwater Flashy stream habitat volume did not differ significantly across seasons (ANOVA *p* = 0.783, Figs. 3, 4).

### Population estimates and capture probability

Simulations showed that population estimates were not biased within the main range of capture probabilities (0.30–0.60) and population sizes (50–300) that we observed. Precision increased as capture probabilities increased from 0.30 to 0.90 and population sizes from 50 to 300 individuals. Simulations indicated that population estimates showed a slight negative bias at capture probabilities of 0.25 and population sizes of 50, and this negative bias increased substantially as capture probabilities decreased. Falling Water during late summer 2002 had the lowest capture probabilities (~ 0.25) and population sizes (~ 50) during this study and therefore the fish population sizes may have been slightly underestimated at this time period and site.

### Fish assemblage structure

We collected over 43,000 fish comprising 30 species from the six headwater streams in 2002 and 2003 (Table 2, Tables S1-S2). Fish species richness and abundance remained relatively stable within streams and across seasons, but densities changed substantially as a result of decreased habitat volume. There were no significant interactions or main effects for total fish abundance (ANOVA *p* ≥ 0.282; Figs. 3, 4). Fish species richness showed no significant interactions (ANOVA *p* ≥ 0.300), but richness differed significantly among seasons (ANOVA *p* = 0.046) with richness greater in autumn than spring (Tukey’s *p* = 0.051; Figs. 3, 4). There was no significant flow regime by season by year interaction for total fish density (ANOVA *p* = 0.784, Figs. 3, 4). However, there was a significant flow regime by season interaction (ANOVA *p* = 0.032, Figs. 3, 4). In Runoff/Intermittent Flashy streams, total fish density differed significantly among seasons (ANOVA *p* = 0.001) with densities significantly greater in autumn than spring and early summer (Tukey’s *p* ≤ 0.009) and greater in summer than spring (Tukey’s *p* = 0.038), whereas Groundwater Flashy stream total fish density did not differ significantly across seasons (ANOVA *p* = 0.604, Figs. 3, 4).

**Table 2:** Fish species collected in 2002 and 2003 and associated life history strategies.

Fish taxa	Common name	Abbreviation	Life history	Characteristic
**Petromyzontidae**				
Lampetra appendix*	American Brook Lamprey	ABL	Equilibrium	
*Lampetra spp.*	Lamprey spp.	LPY	Equilibrium	GF*
Lampetra spp.*	Lamprey ammocoete spp.	AMM	Equilibrium	GF
**Cyprinidae**				
*Campostoma anomalum*	Central Stoneroller	CSR	Periodic	RIF
*Nocomis biguttatus*	Hornyhead Chub	HHC	Periodic	GF
*Notropis boops*	Bigeye Shiner	BES	Opportunistic	
*Notropis galacturus*	Whitetail Shiner	WTS	Opportunistic	RIF
*Notropis nubilus*	Ozark Minnow	OZM	Opportunistic	RIF
*Notropis ozarcanus*	Ozark Shiner	OZS	Opportunistic	
*Notropis pilsbryi*	Duskystripe Shiner	DSS	Opportunistic	GF *
*Notropis telescopus*	Telescope Shiner	TSS	Opportunistic	
*Phoxinus erythrogaster*	Southern Redbelly Dace	SRD	Opportunistic	Both
*Semotilus atromaculatus*	Creek Chub	CRC	Periodic	RIF
**Catostomidae**				
*Hypentelium nigricans*	Northern Hog Sucker	NHS	Periodic	RIF
Moxostoma duquesnei*	Black Redhorse	BRH	Periodic	
*Moxostoma* spp.	redhorse sucker spp.	RH	Periodic	RIF
**Ictaluridae**				
*Noturus exilis*	Slender Madtom	SLM	Opp./Equil	
Noturus flavater**	Checkered Madtom	CKM	Opp./Equil	RIF
**Fundulidae**				
*Fundulus catenatus*	Northern Studfish	NSF	Opportunistic	
*Fundulus olivaceus*	Blackspotted Topminnow	BTM	Opportunistic	GF
**Centrarchidae**				
*Ambloplites constellatus*	Ozark Bass	OZB	Equilibrium	GF**
*Lepomis cyanellus*	Green Sunfish	GSF	Equilibrium	Both**
Lepomis macrochirus**	Bluegill	BLG	Equilibrium	GF**
*Lepomis megalotis*	Longear Sunfish	LES	Equilibrium	Both
*Micropterus dolomieui*	Smallmouth Bass	SMB	Equil./Per	
**Percidae**				
*Etheostoma blennoides*	Greenside Darter	GSD	Opp./Equil	RIF
*Etheostoma caeruleum*	Rainbow Darter	RBD	Opp./Equil	Both
*Etheostoma flabellare*	Fantail Darter	FTD	Equilibrium	GF
Etheostoma juliae*	Yoke Darter	YKD	Opp./Equil	
*Etheostoma punctalatum*	Stippled Darter	STD	Opp./Equil	RIF
*Etheostoma spectabile*	Orangethroat Darter	OTD	Opp./Equil	Both
**Cottidae**				
*Cottus carolinae*	Banded Sculpin	BDS	Equilibrium	GF

Characteristic species were based on high relative densities in Runoff/Intermittent Flashy (RIF), Groundwater Flashy (GF) or both. Life history strategy as in^12^. *only in 2002, **only in 2003.

**Figure 5:**
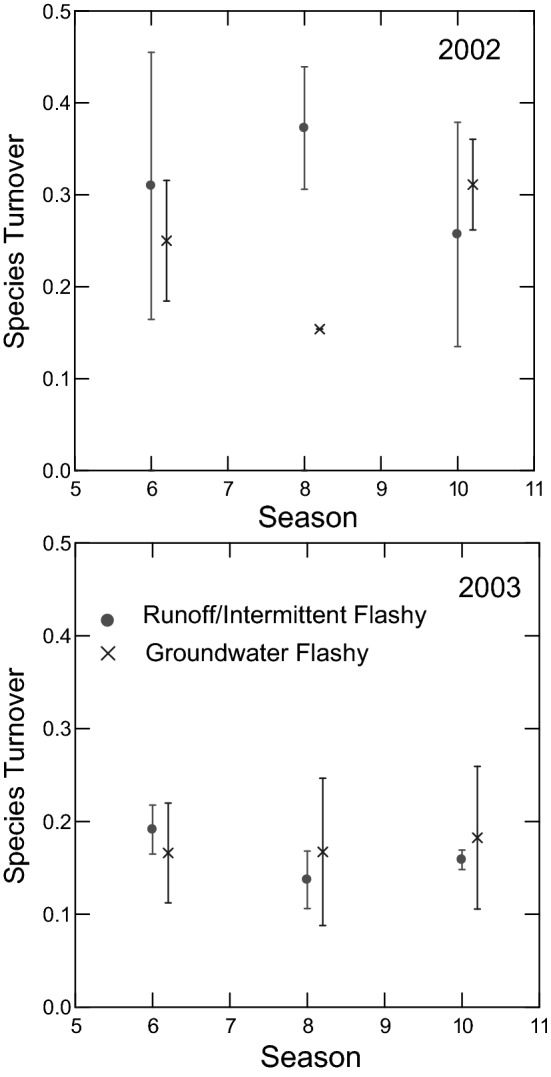
Mean (± SE) seasonal total fish species turnover in Runoff/Intermittent Flashy and Groundwater Flashy streams in 2002 and 2003. Turnover times are spring-early summer = 6, early summer-summer = 8 and summer-autumn = 10. Points are jittered for ease of viewing. Figure produced with SYSTAT 13.

Mixed model analysis showed weak response variable-habitat relationships with strong season effects in 2002, and stronger habitat relationships and no season effect in 2003. In 2002, water depth was the best model of those examined for species richness and total density (Table 3). However, there were no significant relationships between water depth and fish species richness (Table 4), whereas total density showed a significant negative relationship with water depth (Table 4). The best model for total fish abundance was the global model in 2002 (Table 3), but there were no significant relationships between the habitat variables and total abundance (Table 4). Season had a strong effect and was significant in all models in 2002, whereas flow regime was not significant for any model (Table 4). In 2003, the best model for fish species richness was the depth and current velocity model (Table 3). Species richness was positively related to depth and negatively related to current velocity (Table 4). The best model for total fish abundance was the depth model in 2003 (Table 3), with depth being positively related to total abundance (Table 4). The best model for total fish density was the current velocity model in 2003 (Table 3), with velocity being negatively related to total density (Table 4). No response variable showed a significant relationship with season or flow regime in 2003 (Table 4).

**Table 3:** Mixed models ranked by AIC_c_ for three response variables in 2002 and 2003.

Model (by year and response variable)	K	AICc	ΔAICc	w_i_
**2002**				
**Richness**				
Depth + Season + Flow Regime + (1|Stream/Reach)	7	634.27	0.00	0.42
Velocity + Season + Fow Regime + (1|Stream/Reach)	7	636.16	1.90	0.16
Substrate + Season + Flow Regime + (1|Stream/Reach)	7	636.24	1.97	0.16
Depth + Velocity + Season + Flow Regime + (1|Stream/Reach)	8	636.94	2.67	0.11
Depth + Substrate + Season + Flow Regime + (1|Stream/Reach)	8	637.34	3.07	0.09
**Total abundance**				
Depth + Substrate + Velocity + Season + Flow Regime + (1|Stream/Reach)	9	1868.79	0.00	0.90
**Total density**				
Depth + Season + Flow Regime + (1|Stream/Reach)	7	395.24	0.00	0.57
Substrate + Season + Flow Regime + (1|Stream/Reach)	7	398.03	2.79	0.14
Depth + Substrate + Season + Flow Regime + (1|Stream/Reach)	8	398.20	2.96	0.13
Velocity + Season + Fow Regime + (1|Stream/Reach)	7	399.53	4.29	0.07
Depth + Velocity + Season + Flow Regime + (1|Stream/Reach)	8	399.72	4.48	0.06
**2003**				
**Richness**				
Depth + Velocity + Season + Flow Regime + (1|Stream/Reach)	8	654.46	0.00	0.76
Depth + Velocity + Substrate + Season + Flow Regime + (1|Stream/Reach)	9	657.55	3.10	0.16
Depth + Season + Flow Regime + (1|Stream/Reach)	7	659.53	5.07	0.06
**Total abundance**				
Depth + Season + Flow Regime + (1|Stream/Reach)	7	371.88	0.00	0.65
Depth + Velocity + Season + Flow Regime + (1|Stream/Reach)	8	373.92	2.04	0.23
Depth + Substrate + Season + Flow Regime + (1|Stream/Reach)	8	376.12	4.24	0.08
**Total density**				
Velocity + Season + Fow Regime + (1|Stream/Reach)	7	349.33	0.00	0.81
Depth + Velocity* + Season + Flow Regime + (1|Stream/Reach)	8	354.01	4.68	0.08
Substrate + Velocity* + Season + Flow Regime + (1|Stream/Reach)	8	354.52	5.19	0.06

Models with Akaike weights > 0.05 are shown.

**Table 4:** Model averaged parameter estimates, standard error, lower and upper 95% CI by year and response variable.

Year and Response	Parameter	Estimate	SE	Lower	Upper
**2002**					
**Richness**	Intercept	4.95	1.15	2.70	7.21
	Depth	0.23	0.27	− 0.30	0.75
	Substrate	0.03	0.13	− 0.23	0.28
	Velocity	− 0.02	0.14	− 0.30	0.26
	Season*	0.45	0.11	0.22	0.67
	Flow regime	− 0.25	1.10	− 2.40	1.90
**Total abundance**	Intercept	− 137.23	158.98	− 448.83	174.37
	Depth	24.43	26.93	− 28.35	77.21
	Substrate	− 21.00	27.10	− 53.12	32.12
	Velocity	0.20	30.72	− 60.21	60.41
	Season*	49.40	15.50	19.02	79.78
	Flow regime	56.83	157.37	− 251.62	365.28
**Total density**	Intercept	− 0.94	0.59	− 2.10	0.22
	Depth*	− 0.26	0.11	− 0.48	− 0.04
	Season*	0.25	0.04	0.17	0.33
	Flow regime	− 0.19	0.71	− 1.58	1.20
**2003**					
**Richness**	7.12	1.03	5.10	9.14	
	Depth*	0.85	0.19	0.48	1.22
	Velocity*	− 0.60	0.20	− 0.99	− 0.21
	Season	0.08	0.09	− 0.09	0.27
	Flow regime	0.68	1.16	− 1.59	2.95
**Total abundance**	Intercept	6.07	0.39	5.31	6.83
	Depth*	0.29	0.07	0.15	0.43
	Season	− 0.89	0.30	− 1.49	− 0.30
	Flow Regime	− 0.45	0.30	− 1.03	0.13
**Total density**	Intercept	0.79	0.44	− 0.07	1.65
	Velocity*	− 0.21	0.07	− 0.35	− 0.07
	Season	− 0.03	0.03	− 0.03	0.002
	Flow Regime	0.18	0.55	− 0.90	1.26

Model averaging was done for top models (AICc < 2) for each species. Parameter estimates with 95% CI not overlapping zero are considered significant and marked with an asterisk.

**Figure 6:**
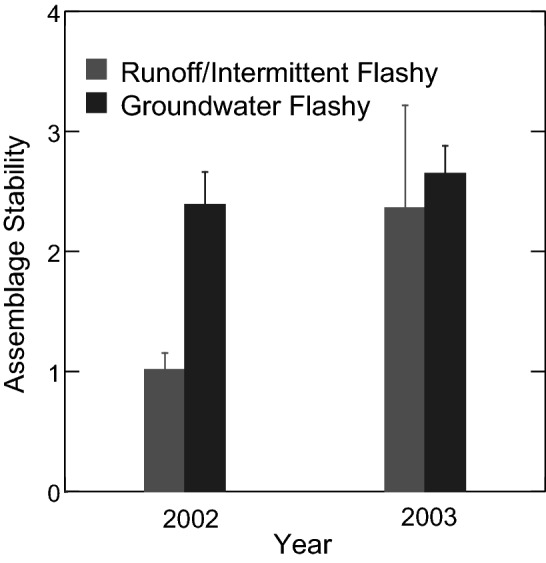
Mean (± SE) assemblage stability in Runoff/Intermittent Flashy and Groundwater Flashy streams in 2002 and 2003. Figure produced with SYSTAT 13.

Species turnover (proportion of species differing between time points) varied by year and assemblage stability (temporal mean divided by temporal standard deviation) varied by year and flow regime. Species turnover showed no two- or three-way interactions among flow regime, season and year (ANOVA *p* > 0.05). There was a strong year effect with species turnover significantly greater in 2002 than 2003 (ANOVA *p* = 0.001, Fig. 5). Assemblage stability showed a marginal flow regime by year interaction (ANOVA *p* = 0.053; Fig. 6). Assemblage stability was significantly lower in 2002 than 2003 in Runoff/Intermittent Flashy streams (ANOVA *p* = 0.049), but did not differ between years in Groundwater Flashy streams (ANOVA *p* = 0.374; Fig. 6). In 2002, assemblage stability was significantly lower in Runoff/Intermittent Flashy than Groundwater Flashy streams (ANOVA *p* = 0.003), whereas in 2003 the two flow regimes did not differ significantly in assemblage stability (ANOVA *p* = 0.537; Fig. 6).

**Figure 7:**
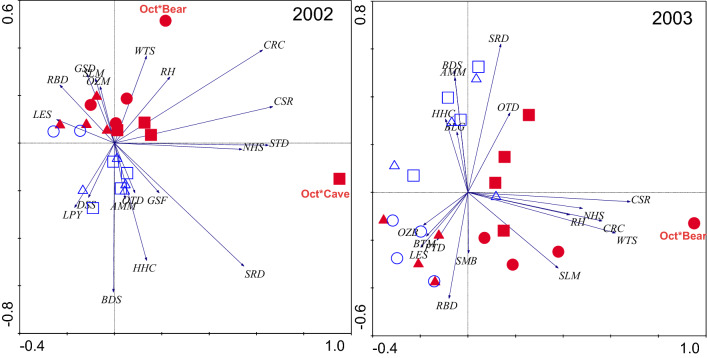
Redundancy analysis examining the relationship between fish species densities and season by stream interactions in 2002 and 2003. Filled symbols (red) denote Runoff/Intermittent Flashy streams and open symbols (blue) denote Groundwater Flashy streams. Arrows represent fish species densities. Fish species abbreviations are as in Table 2. Centroids denote season by stream categories. Runoff/Intermittent Flashy streams are Bear Creek = circles, Cave Creek = Squares, Falling Water Creek = triangles, and Groundwater Flashy streams are Sylamore Creek = circles, Tomahawk Creek = squares, and Water Creek = triangles. Percent variance of species-environment relationships explained was 2002 RDA axis 1 = 78.9%, RDA axis 2 = 19.3%; 2003 RDA axis 1 = 75.9%, RDA axis 2 = 20.8%. Figure produced with CANOCO 4^31^.

Redundancy analysis showed a significant relationship between fish species densities and explanatory variables in both 2002 (Monte Carlo *p* = 0.001) and 2003 (Monte Carlo *p* = 0.001). There was seasonal change in fish assemblage structure (species composition and relative density) that appeared to be driven largely by changes in fish species densities. Fish assemblages showed a separation between flow regimes in 2002 along RDA axis 2 and in 2003 along RDA axis 1 (Fig. 7). In both years, there were greater densities of central stonerollers, creek chubs, whitetail shiners and slender madtoms in Runoff/Intermittent Flashy streams and greater densities of banded sculpins, hornyhead chubs and lampreys in Groundwater Flashy streams (Fig. 7). Southern redbelly dace were abundant in Groundwater Flashy streams, but they were also found in high numbers in Cave Creek, an Intermittent Flashy stream. The other strong pattern found in the RDA ordinations was the seasonal shifts in fish species densities driven by strong increases in densities of the common fish species from spring through autumn, especially in Runoff/Intermittent Flashy streams. In 2002, autumn differed most from the other seasons, with particularly high relative fish densities in Bear and Cave Creeks (Fig. 7). In 2003, there was a strong change in fish species densities in Bear Creek along RDA axis 1 and Cave Creek along RDA axis 2 (Fig. 7).

Opportunistic, Opportunistic/Equilibrium and Equilibrium species were characteristic of both stream types. Periodic and opportunistic species were characteristic of Runoff/Intermittent Flashy streams, whereas mainly equilibrium species were characteristic of Groundwater Flashy streams (Table 2). Only one periodic species was characteristic of Groundwater Flashy streams and four periodic species were characteristic of Runoff/Intermittent Flashy streams. In 2002, three fish species occurred only in Runoff/Intermittent Flashy streams and five fish species occurred only in Groundwater Flashy streams, whereas in 2003 five fish species occurred only in Runoff/Intermittent Flashy streams and seven fish species occurred only in Groundwater Flashy streams (Table 2). In 2002, 20 of 28 fish species occurred in both stream types and in 2003 16 of 28 species occurred in both stream types. Six of the 15 species found only in a single stream type were characteristic species.

## Discussion

Hydrology appeared to play a major role in structuring fish assemblages of Runoff/Intermittent Flashy and Groundwater Flashy streams and the patterns largely followed expectations from ecological theory. In the case of Runoff/Intermittent Flashy streams, highly variable discharge with high flows in spring and extensive stream drying in summer led to strong seasonal changes in fish species densities with reduced assemblage stability but this was dependent on hydrologic variability. In Groundwater Flashy streams, more seasonally stable flows led to less extreme seasonal shifts in fish species densities and greater assemblage stability. Others have shown strong effects of drought on stream fish assemblage dynamics^14,40,41^. Numerous studies have examined the effects of drought and temporal variation related to hydrology on fish assemblage dynamics in streams^42–44^, but few have explicitly examined the role of differing flow regimes in this process. Fish assemblage structure differed among hydrologically variable and hydrologically stable streams in Wisconsin and Minnesota^5^. Warfe et al.^8^ examined three intermittent and three perennial streams in Tasmania and found that they differed in abiotic and biotic variables including productivity and macroinvertebrate assemblage structure, whereas fish assemblages were not strong indicators of flow regime. However, they suggested this was largely due to low diversity and abundance of fish in these systems, which were much lower than species richness and fish abundance in the present study. Another study found that fish assemblage stability (annual variation of fish biomass) was less variable in more species rich communities and was not associated with hydrology^45^. We found that spatial and temporal variation in hydrology appeared to have a strong influence on fish assemblage dynamics in Ozark streams with lower assemblage stability and greater fluctuations in density in more hydrologically variable streams.

Differences in fish assemblages among flow regimes appeared to be responsible for driving some of the patterns we saw in relationships among season and fish assemblage structure. In Runoff/Intermittent Flashy streams mainly periodic and opportunistic species were driving the strong seasonal shifts in density, whereas in Groundwater Flashy streams a set of opportunistic, equilibrium and periodic species influenced the patterns of fish assemblage structure. These differences among seasons in fish assemblage structure were largely due to changes in fish density over time. In Runoff/Intermittent Flashy streams these changes in density were largely influenced by changes in habitat volume. Therefore, it appears that Runoff/Intermittent Flashy streams have a strong concentration effect due to drought and stream drying, whereas Groundwater Flashy streams do not.

This type of concentration effect and boom and bust cycles are common in intermittent streams and are related to stream drying and fish movement. Fish may move among habitats and stream reaches when flows are high and then may be trapped when flows recede^44^. Drying and loss of habitat volume can then produce a concentration effect leading to high fish densities. This can also lead to episodic isolation and habitat fragmentation that can prevent dispersal and rescue effects^46,47^. However, intense drying can lead to substantial fish mortality, including the extreme of extirpation of all individuals within the habitat unit^4,44^. It is also likely that recruitment affected the seasonal density changes in these systems as most of the fish species are spring and summer spawners. In our study streams drying and a concentration effect were important especially during the drought year. However, it is unclear the role that fish movement and recruitment may have played in shaping these patterns and this is a potential avenue for future study.

We found fewer environment-fish relationships during a drought year. Fewer environment-fish relationships during drought could be due to increased biotic interactions. As streams dry during drought, predation often increases and competition is also likely to intensify^48^. Ludlam and Magoulick^49^ found that seasonal drought led to increased effects of fish and crayfish consumers on periphyton biomass in Ozark streams. These increased biotic interactions could lead to reductions in importance of abiotic factors. As expected, we found fish species richness was positively related to water depth during a non-drought year, but this was not the case during a drought year. This suggests that strong drying may negate some environment-fish relationships. However, previous research in Groundwater Flashy streams in the Ozark Highlands found that there were more fish assemblage-environment relationships during a drought year than a wet year, but more macroinvertebrate-environment relationships during the wet year than drought year^17^. It is likely that assemblage-environment relationships are context dependent, influenced by factors such as taxon, flow regime, and drought frequency and intensity.

We found periodic and opportunistic species were characteristic of Runoff/Intermittent Flashy streams, whereas mainly equilibrium species were characteristic of Groundwater Flashy streams. Poff and Allan^5^ found that fish assemblages from hydrologically variable sites tended to be generalist feeders, tolerant of silt and slow-velocity species associated with headwater streams. Olden and Kennard^13^ examined how southeastern USA and eastern Australia fish life history strategies related to hydrologic variability and productivity and found that opportunistic strategists were associated positively and periodic strategists were associated negatively with hydrologic variability. McManamay et al.^50^ related fish traits to hydrologic classes across the conterminous USA and found that periodic strategists were associated with stable, predictable flows and opportunistic strategists were associated with intermittent, variable flows. Our findings agree with others in that opportunistic strategists were associated with hydrologic variability (i.e. Runoff/Intermittent Flashy streams) and equilibrium strategists were associated with hydrologic stability (i.e. Groundwater Flashy streams). However, in contrast to previous studies, we also found periodic strategists to be associated with hydrologically variable streams. Periodic strategists produce large clutches and have high adult survival during periods of suboptimal conditions^12^. Therefore, it may not be surprising that these species were associated with Runoff/Intermittent Flashy streams that had flooding in spring and strong drying during summer and autumn. Additionally, we did a simple descriptive examination of traits and did not account for phylogeny, so it is possible that some of the differences among studies relates to phylogeny. However, this seems unlikely given that we found similar patterns to others for two of three life history strategies.

Unexpectedly, fish species richness was generally temporally stable and both species richness and seasonal turnover did not differ between flow regimes. However, seasonal species turnover varied strongly by year and appeared strongly affected by temporal variation in hydrology. In particular, the drought year significantly increased species turnover. Fish assemblage stability was also reduced in the drought year in Runoff/Intermittent Flashy streams and was lower in Runoff/Intermittent Flashy than Groundwater Flashy streams in the drought year. Therefore, although drought and flow regime may not have strong effects on fish species richness, species composition and assemblage stability appear to be affected by drought. Based on previous work examining survival and movement in similar systems, we suspect that increased mortality during drought may lead to species extirpations, but emigration may also play a role^51^, and both of these are likely to lead to increased species turnover and reduced assemblage stability. In Mediterranean streams, Magalhaes et al.^52^ found that multi-year droughts had little effect on fish species richness, composition and rank abundances, but did affect individual species abundances. Others have suggested that fish assemblages recover rapidly from drought^4,52,53^. In experimental manipulations of drought and fragmentation, Driver and Hoeinghaus^54^ found that fish survivorship and species diversity were affected little at the experimental unit level, but effects were found at the habitat level. This suggests that effects of drought are likely to be dependent on spatial and temporal scale. Future research would be required to determine mechanisms behind these changes in fish assemblage structure.

Differences in capture probabilities and species detection probabilities among seasons and streams could potentially explain some changes in fish density. However, our species detection probabilities were high and relatively stable and our simulations showed that changes from 0.3 to 0.9 in capture probabilities among sampling periods will lead to increased precision of estimates for population sizes 50–300, but estimates should be unbiased. The only exception to this could be Falling Water during late summer 2002, where we may have slightly underestimated abundances at this time. However, this should not have had a noticeable effect on the overall estimates for Runoff/Intermittent Flashy streams. Therefore, changes in fish densities and species richness over time in some of our study streams were real and not simply the result of changes in species detection or capture probabilities.

It is likely that the fish assemblage dynamics we observed could be influenced by factors that alter stream hydrology, such as climate change or water withdrawals. Water withdrawals could lead to increased flow variability, frequency of low flows and zero flow days, and fall rate, while decreasing base flow and minimum 30-day mean flow (Leasure and Magoulick unpublished data). Future work addressing how alteration of hydrologic variation will affect fish and other biotic assemblages would be worthwhile.

## Conclusions

We found that spatial and temporal variation in hydrology had a strong influence on fish assemblage dynamics in Ozark streams with lower assemblage stability and greater fluctuations in density in more hydrologically variable streams and years. We found fewer environment-fish relationships during seasonal drying in a drought year. Fish species life history strategies generally followed expected patterns, but periodic strategists were unexpectedly associated with more hydrologically variable systems. Understanding relationships between fish assemblage structure and hydrologic variation is vital for conservation of fish biodiversity. Future work should consider addressing how alteration of hydrologic variation will affect biotic assemblages.

## Supplementary Information

Supplementary Tables.
