# Supplementary material for: Hydrologic variation influences stream fish assemblage dynamics through flow regime and drought
Source: Sci Rep. 2021 May 21;11:10704. doi: 10.1038/s41598-021-89632-3 (PMC8140097; doi:10.1038/s41598-021-89632-3)
Supplement: Supplementary file 1 — Supplementary Tables. [file 41598_2021_89632_MOESM1_ESM.docx]

Table S1. Mean depth (cm), current velocity (cm/s), substrate size category, total fish abundance, total fish density (inds/m2) and species densities by season and stream in 2002. Fish species abbreviations are as in Table 2.

| SEASON | STREAM | DEPTH | VELOCITY | SUBSTRATE | TOTAL ABUNDANCE | TOTAL DENSITY | BES | CRC | CSR |
| --- | --- | --- | --- | --- | --- | --- | --- | --- | --- |
| April | Bear | 32.37 | 17.30 | 4.87 | 103.83 | 0.435 | 0.0010 | 0.0110 | 0.2071 |
| April | Cave | 52.07 | 14.48 | 4.74 | 163.60 | 0.376 | 0.0000 | 0.0581 | 0.1893 |
| April | Falling Water | 34.48 | 20.00 | 4.65 | 20.75 | 0.087 | 0.0000 | 0.0031 | 0.0300 |
| April | Tomahawk | 29.35 | 15.72 | 4.33 | 105.25 | 0.614 | 0.0000 | 0.0026 | 0.0139 |
| April | Water | 22.82 | 11.86 | 3.65 | 209.00 | 0.865 | 0.0000 | 0.0007 | 0.0927 |
| June | Bear | 23.77 | 5.88 | 5.08 | 34.17 | 0.190 | 0.0000 | 0.0230 | 0.0000 |
| June | Cave | 33.74 | 5.03 | 4.41 | 512.00 | 1.889 | 0.0000 | 0.1129 | 0.8520 |
| June | Falling Water | 25.96 | 6.89 | 4.83 | 76.86 | 0.599 | 0.0000 | 0.0158 | 0.1404 |
| June | Tomahawk | 19.59 | 2.67 | 4.31 | 225.13 | 1.648 | 0.0000 | 0.0011 | 0.1717 |
| June | Water | 15.97 | 4.84 | 3.73 | 299.14 | 1.590 | 0.0000 | 0.0026 | 0.2653 |
| August | Bear | 20.74 | 3.31 | 4.89 | 239.50 | 1.708 | 0.0000 | 0.1613 | 0.9005 |
| August | Cave | 22.58 | 1.83 | 4.37 | 515.60 | 2.057 | 0.0000 | 0.1829 | 1.0943 |
| August | Falling Water | 20.48 | 5.18 | 4.85 | 81.50 | 0.607 | 0.0000 | 0.0168 | 0.1704 |
| August | Sylamore | 29.11 | 4.81 | 4.02 | 124.20 | 0.409 | 0.0018 | 0.0000 | 0.0459 |
| August | Tomahawk | 19.88 | 1.48 | 4.34 | 314.75 | 2.165 | 0.0000 | 0.0021 | 0.6496 |
| August | Water | 13.22 | 2.73 | 3.97 | 425.86 | 2.021 | 0.0000 | 0.0151 | 0.5437 |
| October | Bear | 9.27 | 0.02 | 4.88 | 314.17 | 3.538 | 0.0000 | 0.5552 | 2.4485 |
| October | Cave | 12.44 | 0.00 | 4.42 | 1230.33 | 8.857 | 0.0000 | 0.7425 | 4.6339 |
| October | Falling Water | 11.70 | 0.26 | 4.63 | 130.63 | 1.325 | 0.0000 | 0.1286 | 0.3973 |
| October | Sylamore | 20.88 | 1.83 | 4.01 | 212.00 | 0.659 | 0.0000 | 0.0000 | 0.2866 |
| October | Tomahawk | 20.21 | 1.27 | 4.16 | 305.38 | 2.371 | 0.0000 | 0.0000 | 0.6160 |
| October | Water | 13.55 | 1.94 | 3.75 | 400.50 | 1.958 | 0.0000 | 0.0232 | 0.5269 |

| SEASON | STREAM | DSS | GSD | GSF | LES | OTD | OZM | RBD | SLM | SRD |
| --- | --- | --- | --- | --- | --- | --- | --- | --- | --- | --- |
| April | Bear | 0.0072 | 0.0114 | 0.0008 | 0.0023 | 0.1191 | 0.0008 | 0.0220 | 0.0072 | 0.0016 |
| April | Cave | 0.0021 | 0.0003 | 0.0000 | 0.0000 | 0.0275 | 0.0000 | 0.0025 | 0.0085 | 0.0819 |
| April | Falling Water | 0.0000 | 0.0045 | 0.0000 | 0.0019 | 0.0141 | 0.0007 | 0.0157 | 0.0053 | 0.0000 |
| April | Tomahawk | 0.0542 | 0.0000 | 0.0058 | 0.0000 | 0.0975 | 0.0000 | 0.0000 | 0.0009 | 0.2802 |
| April | Water | 0.0951 | 0.0000 | 0.0000 | 0.0000 | 0.2126 | 0.0018 | 0.0000 | 0.0015 | 0.2766 |
| June | Bear | 0.0247 | 0.0202 | 0.0000 | 0.0000 | 0.0000 | 0.0016 | 0.0000 | 0.0162 | 0.0225 |
| June | Cave | 0.0249 | 0.0004 | 0.0008 | 0.0008 | 0.1086 | 0.0000 | 0.0334 | 0.0155 | 0.7447 |
| June | Falling Water | 0.0586 | 0.0623 | 0.0005 | 0.0025 | 0.0833 | 0.0448 | 0.1359 | 0.0317 | 0.0000 |
| June | Tomahawk | 0.1130 | 0.0000 | 0.0082 | 0.0005 | 0.1807 | 0.0000 | 0.0000 | 0.0037 | 0.9033 |
| June | Water | 0.1275 | 0.0000 | 0.0000 | 0.0000 | 0.2128 | 0.0009 | 0.0017 | 0.0069 | 0.9017 |
| August | Bear | 0.0246 | 0.0441 | 0.0000 | 0.0000 | 0.2031 | 0.0150 | 0.0365 | 0.0516 | 0.1267 |
| August | Cave | 0.0802 | 0.0002 | 0.0040 | 0.0007 | 0.0406 | 0.0000 | 0.0000 | 0.0184 | 0.4598 |
| August | Falling Water | 0.1396 | 0.0255 | 0.0000 | 0.0000 | 0.1069 | 0.0000 | 0.0461 | 0.0199 | 0.0000 |
| August | Sylamore | 0.1639 | 0.0000 | 0.0000 | 0.0134 | 0.0014 | 0.0029 | 0.0699 | 0.0359 | 0.0000 |
| August | Tomahawk | 0.1028 | 0.0000 | 0.0050 | 0.0000 | 0.1270 | 0.0000 | 0.0000 | 0.0000 | 0.6947 |
| August | Water | 0.2768 | 0.0000 | 0.0000 | 0.0000 | 0.2469 | 0.0017 | 0.0000 | 0.0222 | 0.8067 |
| October | Bear | 0.0258 | 0.0290 | 0.0000 | 0.0008 | 0.2009 | 0.0285 | 0.0060 | 0.0278 | 0.0446 |
| October | Cave | 0.2318 | 0.0022 | 0.0109 | 0.0000 | 0.0862 | 0.0000 | 0.0000 | 0.0409 | 2.6972 |
| October | Falling Water | 0.3647 | 0.0593 | 0.0000 | 0.0169 | 0.1401 | 0.0255 | 0.0709 | 0.0934 | 0.0000 |
| October | Sylamore | 0.2628 | 0.0000 | 0.0000 | 0.0276 | 0.0000 | 0.0033 | 0.1327 | 0.0224 | 0.0000 |
| October | Tomahawk | 0.2275 | 0.0000 | 0.0062 | 0.0000 | 0.1915 | 0.0081 | 0.0000 | 0.0000 | 0.9145 |
| October | Water | 0.3090 | 0.0000 | 0.0000 | 0.0000 | 0.1674 | 0.0018 | 0.0000 | 0.0100 | 0.6857 |

| SEASON | STREAM | STD | WTS | BDS | HHC | NHS | SMB | AMM | BTM | NSF |
| --- | --- | --- | --- | --- | --- | --- | --- | --- | --- | --- |
| April | Bear | 0.0008 | 0.0036 | 0.0000 | 0.0000 | 0.0000 | 0.0000 | 0.0000 | 0.0000 | 0.0000 |
| April | Cave | 0.0028 | 0.0000 | 0.0006 | 0.0013 | 0.0009 | 0.0008 | 0.0000 | 0.0000 | 0.0000 |
| April | Falling Water | 0.0000 | 0.0005 | 0.0000 | 0.0000 | 0.0005 | 0.0000 | 0.0000 | 0.0000 | 0.0000 |
| April | Tomahawk | 0.0000 | 0.0000 | 0.1058 | 0.0247 | 0.0048 | 0.0000 | 0.0006 | 0.0000 | 0.0000 |
| April | Water | 0.0000 | 0.0000 | 0.0596 | 0.0423 | 0.0000 | 0.0000 | 0.0000 | 0.0000 | 0.0028 |
| June | Bear | 0.0030 | 0.0000 | 0.0000 | 0.0000 | 0.0000 | 0.0000 | 0.0000 | 0.0000 | 0.0000 |
| June | Cave | 0.0335 | 0.0000 | 0.0095 | 0.0041 | 0.0000 | 0.0000 | 0.0000 | 0.0000 | 0.0021 |
| June | Falling Water | 0.0000 | 0.0055 | 0.0000 | 0.0000 | 0.0005 | 0.0011 | 0.0000 | 0.0000 | 0.0000 |
| June | Tomahawk | 0.0000 | 0.0000 | 0.1596 | 0.0404 | 0.0052 | 0.0000 | 0.0165 | 0.0000 | 0.0000 |
| June | Water | 0.0000 | 0.0000 | 0.1628 | 0.1126 | 0.0000 | 0.0000 | 0.0066 | 0.0000 | 0.0000 |
| August | Bear | 0.0071 | 0.0867 | 0.0000 | 0.0000 | 0.0000 | 0.0000 | 0.0000 | 0.0000 | 0.0000 |
| August | Cave | 0.0503 | 0.0000 | 0.0056 | 0.0213 | 0.0034 | 0.0000 | 0.0000 | 0.0000 | 0.0000 |
| August | Falling Water | 0.0005 | 0.0028 | 0.0000 | 0.0000 | 0.0000 | 0.0014 | 0.0000 | 0.0000 | 0.0000 |
| August | Sylamore | 0.0000 | 0.0000 | 0.0356 | 0.0029 | 0.0000 | 0.0045 | 0.0000 | 0.0022 | 0.0028 |
| August | Tomahawk | 0.0000 | 0.0000 | 0.1611 | 0.0471 | 0.0033 | 0.0000 | 0.0026 | 0.0000 | 0.0000 |
| August | Water | 0.0000 | 0.0000 | 0.0939 | 0.0904 | 0.0000 | 0.0000 | 0.0036 | 0.0000 | 0.0000 |
| October | Bear | 0.0008 | 0.1947 | 0.0000 | 0.0000 | 0.0131 | 0.0000 | 0.0000 | 0.0000 | 0.0000 |
| October | Cave | 0.1018 | 0.0000 | 0.0171 | 0.0785 | 0.0396 | 0.0022 | 0.0000 | 0.0000 | 0.0000 |
| October | Falling Water | 0.0000 | 0.0045 | 0.0000 | 0.0053 | 0.0025 | 0.0026 | 0.0000 | 0.0000 | 0.0000 |
| October | Sylamore | 0.0000 | 0.0000 | 0.0586 | 0.0089 | 0.0005 | 0.0049 | 0.0000 | 0.0147 | 0.0089 |
| October | Tomahawk | 0.0000 | 0.0000 | 0.1363 | 0.0525 | 0.0084 | 0.0000 | 0.0000 | 0.0000 | 0.0000 |
| October | Water | 0.0000 | 0.0000 | 0.1078 | 0.1127 | 0.0000 | 0.0000 | 0.0000 | 0.0000 | 0.0038 |

| SEASON | STREAM | OZB | OZS | ABL | RH | FTD | TSS | YOM | YKD | LPY |
| --- | --- | --- | --- | --- | --- | --- | --- | --- | --- | --- |
| April | Bear | 0.0000 | 0.0000 | 0.0000 | 0.0000 | 0.0000 | 0.0000 | 0.0000 | 0.0000 | 0.0000 |
| April | Cave | 0.0000 | 0.0000 | 0.0000 | 0.0000 | 0.0000 | 0.0000 | 0.0000 | 0.0000 | 0.0000 |
| April | Falling Water | 0.0000 | 0.0000 | 0.0000 | 0.0000 | 0.0000 | 0.0000 | 0.0000 | 0.0000 | 0.0000 |
| April | Tomahawk | 0.0000 | 0.0000 | 0.0000 | 0.0055 | 0.0000 | 0.0000 | 0.0000 | 0.0000 | 0.0000 |
| April | Water | 0.0011 | 0.0000 | 0.0012 | 0.0000 | 0.0000 | 0.0000 | 0.0000 | 0.0000 | 0.0000 |
| June | Bear | 0.0000 | 0.0000 | 0.0000 | 0.0000 | 0.0000 | 0.0104 | 0.0346 | 0.0000 | 0.0000 |
| June | Cave | 0.0000 | 0.0000 | 0.0000 | 0.0000 | 0.0000 | 0.0000 | 0.0210 | 0.0037 | 0.0000 |
| June | Falling Water | 0.0000 | 0.0000 | 0.0000 | 0.0000 | 0.0000 | 0.0000 | 0.0000 | 0.0000 | 0.0000 |
| June | Tomahawk | 0.0000 | 0.0000 | 0.0000 | 0.0000 | 0.0000 | 0.0000 | 0.0032 | 0.0000 | 0.0000 |
| June | Water | 0.0064 | 0.0000 | 0.0000 | 0.0000 | 0.0000 | 0.0000 | 0.0000 | 0.0000 | 0.0000 |
| August | Bear | 0.0000 | 0.0000 | 0.0000 | 0.0016 | 0.0000 | 0.0000 | 0.0000 | 0.0000 | 0.0000 |
| August | Cave | 0.0000 | 0.0000 | 0.0000 | 0.0000 | 0.0000 | 0.0000 | 0.0000 | 0.0000 | 0.0000 |
| August | Falling Water | 0.0000 | 0.0000 | 0.0000 | 0.0000 | 0.0000 | 0.0000 | 0.0000 | 0.0000 | 0.0000 |
| August | Sylamore | 0.0119 | 0.0000 | 0.0005 | 0.0000 | 0.0287 | 0.0006 | 0.0000 | 0.0000 | 0.0000 |
| August | Tomahawk | 0.0000 | 0.0000 | 0.0000 | 0.0000 | 0.0000 | 0.0000 | 0.0009 | 0.0000 | 0.0000 |
| August | Water | 0.0044 | 0.0000 | 0.0000 | 0.0000 | 0.0000 | 0.0000 | 0.0030 | 0.0000 | 0.0000 |
| October | Bear | 0.0000 | 0.0000 | 0.0000 | 0.0113 | 0.0000 | 0.0000 | 0.0000 | 0.0000 | 0.0000 |
| October | Cave | 0.0000 | 0.0000 | 0.0000 | 0.0044 | 0.0000 | 0.0000 | 0.0000 | 0.0000 | 0.0000 |
| October | Falling Water | 0.0000 | 0.0000 | 0.0000 | 0.0005 | 0.0000 | 0.0000 | 0.0023 | 0.0000 | 0.0000 |
| October | Sylamore | 0.0096 | 0.0000 | 0.0000 | 0.0000 | 0.0141 | 0.0031 | 0.0000 | 0.0000 | 0.0021 |
| October | Tomahawk | 0.0000 | 0.0000 | 0.0000 | 0.0000 | 0.0000 | 0.0000 | 0.0000 | 0.0000 | 0.0041 |
| October | Water | 0.0063 | 0.0000 | 0.0000 | 0.0000 | 0.0000 | 0.0000 | 0.0000 | 0.0000 | 0.0130 |

Table S2. Mean depth (cm), current velocity (cm/s), substrate size category, total fish abundance, total fish density (inds/m2) and species densities by season and stream in 2002. Fish species abbreviations are as in Table 2.

| SEASON | STREAM | DEPTH | VELOCITY | SUBSTRATE | TOTAL ABUNDANCE | TOTAL DENSITY | CRC | CSR | DSS |
| --- | --- | --- | --- | --- | --- | --- | --- | --- | --- |
| April | Bear | 27.20 | 8.83 | 4.79 | 134.17 | 0.685 | 0.0412 | 0.4016 | 0.0107 |
| April | Cave | 35.35 | 4.68 | 4.37 | 394.00 | 1.180 | 0.0664 | 0.4042 | 0.0040 |
| April | Falling Water | 25.71 | 8.61 | 4.84 | 70.86 | 0.336 | 0.0158 | 0.1279 | 0.0436 |
| April | Sylamore | 21.53 | 5.14 | 3.85 | 75.25 | 0.168 | 0.0000 | 0.0146 | 0.0204 |
| April | Tomahawk | 20.26 | 2.66 | 4.36 | 174.38 | 1.325 | 0.0000 | 0.2764 | 0.0766 |
| April | Water | 13.61 | 3.27 | 3.98 | 218.17 | 0.980 | 0.0035 | 0.4308 | 0.0732 |
| June | Bear | 25.32 | 8.33 | 4.88 | 187.43 | 1.035 | 0.0816 | 0.6679 | 0.0144 |
| June | Cave | 32.89 | 5.15 | 4.26 | 346.20 | 1.066 | 0.0643 | 0.3823 | 0.0119 |
| June | Falling Water | 27.73 | 10.97 | 5.04 | 66.86 | 0.379 | 0.0118 | 0.0998 | 0.0302 |
| June | Sylamore | 24.50 | 4.78 | 3.73 | 139.00 | 0.479 | 0.0000 | 0.1163 | 0.0576 |
| June | Tomahawk | 23.85 | 7.68 | 4.09 | 230.11 | 1.680 | 0.0000 | 0.3929 | 0.0429 |
| June | Water | 17.65 | 5.78 | 3.55 | 208.29 | 1.224 | 0.0037 | 0.1710 | 0.1153 |
| August | Bear | 17.31 | 2.01 | 4.71 | 204.29 | 1.686 | 0.0573 | 1.1129 | 0.0714 |
| August | Cave | 16.69 | 0.60 | 4.19 | 303.80 | 1.281 | 0.1149 | 0.7666 | 0.0455 |
| August | Falling Water | 17.39 | 1.15 | 4.97 | 96.57 | 0.804 | 0.0587 | 0.2265 | 0.1036 |
| August | Sylamore | 21.37 | 2.82 | 3.64 | 96.60 | 0.333 | 0.0000 | 0.1094 | 0.0804 |
| August | Tomahawk | 18.52 | 1.31 | 4.41 | 143.25 | 1.112 | 0.0000 | 0.5955 | 0.0516 |
| August | Water | 12.03 | 1.24 | 3.68 | 384.50 | 1.997 | 0.0064 | 0.4160 | 0.1237 |
| October | Bear | 12.78 | 0.73 | 4.69 | 277.14 | 3.308 | 0.1469 | 1.9000 | 0.1499 |
| October | Cave | 21.55 | 0.04 | 4.20 | 286.33 | 1.876 | 0.2687 | 1.1145 | 0.0631 |
| October | Falling Water | 14.82 | 0.22 | 4.81 | 48.71 | 0.358 | 0.0303 | 0.1173 | 0.0597 |
| October | Sylamore | 17.57 | 2.45 | 3.84 | 140.50 | 0.442 | 0.0015 | 0.1892 | 0.1155 |
| October | Tomahawk | 21.74 | 4.78 | 4.05 | 95.75 | 0.648 | 0.0000 | 0.4647 | 0.0844 |
| October | Water | 11.67 | 1.43 | 3.82 | 103.17 | 0.556 | 0.0080 | 0.1167 | 0.0808 |

| SEASON | STREAM | GSD | LES | NHS | OTD | OZM | RBD | SLM | WTS | BDS |
| --- | --- | --- | --- | --- | --- | --- | --- | --- | --- | --- |
| April | Bear | 0.0069 | 0.0023 | 0.0015 | 0.0511 | 0.0084 | 0.0038 | 0.0137 | 0.0221 | 0.0000 |
| April | Cave | 0.0000 | 0.0000 | 0.0018 | 0.0758 | 0.0000 | 0.0206 | 0.0112 | 0.0000 | 0.0013 |
| April | Falling Water | 0.0236 | 0.0012 | 0.0024 | 0.0218 | 0.0030 | 0.0206 | 0.0285 | 0.0030 | 0.0000 |
| April | Sylamore | 0.0000 | 0.0082 | 0.0017 | 0.0000 | 0.0006 | 0.0472 | 0.0058 | 0.0000 | 0.0210 |
| April | Tomahawk | 0.0000 | 0.0000 | 0.0053 | 0.2147 | 0.0009 | 0.0000 | 0.0009 | 0.0000 | 0.1074 |
| April | Water | 0.0000 | 0.0000 | 0.0000 | 0.2907 | 0.0000 | 0.0110 | 0.0014 | 0.0000 | 0.0891 |
| June | Bear | 0.0196 | 0.0015 | 0.0008 | 0.1231 | 0.0023 | 0.0053 | 0.0378 | 0.0098 | 0.0000 |
| June | Cave | 0.0000 | 0.0000 | 0.0000 | 0.0589 | 0.0000 | 0.0000 | 0.0078 | 0.0000 | 0.0087 |
| June | Falling Water | 0.0243 | 0.0033 | 0.0000 | 0.0413 | 0.0013 | 0.0466 | 0.0144 | 0.0026 | 0.0000 |
| June | Sylamore | 0.0000 | 0.0118 | 0.0000 | 0.0000 | 0.0051 | 0.0515 | 0.0093 | 0.0000 | 0.0340 |
| June | Tomahawk | 0.0000 | 0.0008 | 0.0000 | 0.1508 | 0.0008 | 0.0000 | 0.0008 | 0.0000 | 0.2199 |
| June | Water | 0.0000 | 0.0000 | 0.0000 | 0.1324 | 0.0000 | 0.0000 | 0.0030 | 0.0000 | 0.1101 |
| August | Bear | 0.0162 | 0.0022 | 0.0043 | 0.0422 | 0.0054 | 0.0281 | 0.0216 | 0.0476 | 0.0000 |
| August | Cave | 0.0008 | 0.0000 | 0.0000 | 0.0162 | 0.0000 | 0.0000 | 0.0100 | 0.0000 | 0.0039 |
| August | Falling Water | 0.0578 | 0.0009 | 0.0009 | 0.0303 | 0.0431 | 0.0229 | 0.0348 | 0.0028 | 0.0000 |
| August | Sylamore | 0.0000 | 0.0257 | 0.0000 | 0.0000 | 0.0013 | 0.0395 | 0.0105 | 0.0000 | 0.0204 |
| August | Tomahawk | 0.0000 | 0.0000 | 0.0011 | 0.0623 | 0.0043 | 0.0000 | 0.0011 | 0.0000 | 0.0634 |
| August | Water | 0.0000 | 0.0000 | 0.0000 | 0.1373 | 0.0000 | 0.0000 | 0.0024 | 0.0000 | 0.0538 |
| October | Bear | 0.0103 | 0.0000 | 0.0147 | 0.0749 | 0.0279 | 0.0132 | 0.0426 | 0.1146 | 0.0000 |
| October | Cave | 0.0000 | 0.0000 | 0.0000 | 0.0117 | 0.0000 | 0.0000 | 0.0210 | 0.0000 | 0.0047 |
| October | Falling Water | 0.0081 | 0.0051 | 0.0020 | 0.0243 | 0.0243 | 0.0081 | 0.0121 | 0.0020 | 0.0000 |
| October | Sylamore | 0.0000 | 0.0089 | 0.0000 | 0.0000 | 0.0007 | 0.0216 | 0.0134 | 0.0000 | 0.0290 |
| October | Tomahawk | 0.0000 | 0.0000 | 0.0011 | 0.0310 | 0.0096 | 0.0000 | 0.0000 | 0.0000 | 0.0395 |
| October | Water | 0.0000 | 0.0000 | 0.0000 | 0.0360 | 0.0000 | 0.0104 | 0.0016 | 0.0000 | 0.0208 |

| SEASON | STREAM | GSF | HHC | SMB | SRD | STD | AMM | OZB | BTM | FTD |
| --- | --- | --- | --- | --- | --- | --- | --- | --- | --- | --- |
| April | Bear | 0.0008 | 0.0114 | 0.0000 | 0.0191 | 0.0000 | 0.0000 | 0.0000 | 0.0000 | 0.0000 |
| April | Cave | 0.0009 | 0.0031 | 0.0009 | 0.2516 | 0.0058 | 0.0000 | 0.0000 | 0.0000 | 0.0000 |
| April | Falling Water | 0.0000 | 0.0000 | 0.0000 | 0.0000 | 0.0000 | 0.0000 | 0.0000 | 0.0000 | 0.0000 |
| April | Sylamore | 0.0000 | 0.0076 | 0.0047 | 0.0000 | 0.0000 | 0.0000 | 0.0023 | 0.0012 | 0.0093 |
| April | Tomahawk | 0.0044 | 0.0141 | 0.0000 | 0.3767 | 0.0000 | 0.0018 | 0.0000 | 0.0000 | 0.0000 |
| April | Water | 0.0000 | 0.0110 | 0.0000 | 0.1208 | 0.0000 | 0.0028 | 0.0014 | 0.0000 | 0.0000 |
| June | Bear | 0.0015 | 0.0008 | 0.0000 | 0.0748 | 0.0008 | 0.0000 | 0.0000 | 0.0000 | 0.0000 |
| June | Cave | 0.0005 | 0.0087 | 0.0000 | 0.2865 | 0.0196 | 0.0000 | 0.0000 | 0.0000 | 0.0000 |
| June | Falling Water | 0.0000 | 0.0007 | 0.0000 | 0.0000 | 0.0000 | 0.0000 | 0.0000 | 0.0000 | 0.0000 |
| June | Sylamore | 0.0000 | 0.0057 | 0.0021 | 0.0000 | 0.0000 | 0.0010 | 0.0093 | 0.0046 | 0.0530 |
| June | Tomahawk | 0.0008 | 0.0056 | 0.0000 | 0.7866 | 0.0000 | 0.0079 | 0.0000 | 0.0000 | 0.0000 |
| June | Water | 0.0000 | 0.0275 | 0.0000 | 0.4997 | 0.0000 | 0.0082 | 0.0007 | 0.0000 | 0.0000 |
| August | Bear | 0.0054 | 0.0065 | 0.0011 | 0.1146 | 0.0022 | 0.0000 | 0.0000 | 0.0000 | 0.0000 |
| August | Cave | 0.0201 | 0.0116 | 0.0000 | 0.1627 | 0.0262 | 0.0000 | 0.0000 | 0.0000 | 0.0000 |
| August | Falling Water | 0.0000 | 0.0028 | 0.0165 | 0.0000 | 0.0000 | 0.0000 | 0.0000 | 0.0000 | 0.0000 |
| August | Sylamore | 0.0000 | 0.0119 | 0.0046 | 0.0000 | 0.0000 | 0.0000 | 0.0099 | 0.0053 | 0.0310 |
| August | Tomahawk | 0.0000 | 0.0226 | 0.0000 | 0.6170 | 0.0000 | 0.0021 | 0.0011 | 0.0000 | 0.0000 |
| August | Water | 0.0000 | 0.0538 | 0.0000 | 0.5838 | 0.0000 | 0.0096 | 0.0016 | 0.0000 | 0.0000 |
| October | Bear | 0.0029 | 0.0044 | 0.0088 | 0.1602 | 0.0000 | 0.0000 | 0.0000 | 0.0000 | 0.0000 |
| October | Cave | 0.0304 | 0.0093 | 0.0023 | 0.5327 | 0.0140 | 0.0000 | 0.0000 | 0.0000 | 0.0000 |
| October | Falling Water | 0.0000 | 0.0000 | 0.0051 | 0.0000 | 0.0000 | 0.0000 | 0.0010 | 0.0000 | 0.0000 |
| October | Sylamore | 0.0000 | 0.0112 | 0.0030 | 0.0000 | 0.0000 | 0.0000 | 0.0037 | 0.0074 | 0.0082 |
| October | Tomahawk | 0.0011 | 0.0150 | 0.0000 | 0.1923 | 0.0000 | 0.0053 | 0.0000 | 0.0000 | 0.0000 |
| October | Water | 0.0000 | 0.0384 | 0.0000 | 0.1879 | 0.0000 | 0.0008 | 0.0000 | 0.0000 | 0.0000 |

| SEASON | STREAM | NSF | TSS | RH | BLG | BES | CKM |
| --- | --- | --- | --- | --- | --- | --- | --- |
| April | Bear | 0.0000 | 0.0000 | 0.0008 | 0.0000 | 0.0000 | 0.0000 |
| April | Cave | 0.0000 | 0.0000 | 0.0000 | 0.0000 | 0.0000 | 0.0000 |
| April | Falling Water | 0.0000 | 0.0000 | 0.0000 | 0.0000 | 0.0000 | 0.0000 |
| April | Sylamore | 0.0041 | 0.0006 | 0.0000 | 0.0000 | 0.0000 | 0.0000 |
| April | Tomahawk | 0.0000 | 0.0000 | 0.0000 | 0.0000 | 0.0000 | 0.0000 |
| April | Water | 0.0000 | 0.0000 | 0.0000 | 0.0000 | 0.0000 | 0.0000 |
| June | Bear | 0.0000 | 0.0000 | 0.0000 | 0.0000 | 0.0015 | 0.0000 |
| June | Cave | 0.0000 | 0.0000 | 0.0000 | 0.0000 | 0.0000 | 0.0000 |
| June | Falling Water | 0.0000 | 0.0000 | 0.0000 | 0.0000 | 0.0000 | 0.0000 |
| June | Sylamore | 0.0036 | 0.0026 | 0.0000 | 0.0000 | 0.0000 | 0.0010 |
| June | Tomahawk | 0.0000 | 0.0000 | 0.0000 | 0.0040 | 0.0000 | 0.0000 |
| June | Water | 0.0000 | 0.0000 | 0.0000 | 0.0000 | 0.0000 | 0.0000 |
| August | Bear | 0.0000 | 0.0000 | 0.0032 | 0.0000 | 0.0000 | 0.0000 |
| August | Cave | 0.0000 | 0.0000 | 0.0000 | 0.0000 | 0.0000 | 0.0000 |
| August | Falling Water | 0.0000 | 0.0000 | 0.0000 | 0.0000 | 0.0000 | 0.0000 |
| August | Sylamore | 0.0040 | 0.0040 | 0.0000 | 0.0000 | 0.0000 | 0.0000 |
| August | Tomahawk | 0.0000 | 0.0000 | 0.0000 | 0.0021 | 0.0000 | 0.0000 |
| August | Water | 0.0016 | 0.0000 | 0.0000 | 0.0000 | 0.0000 | 0.0000 |
| October | Bear | 0.0000 | 0.0000 | 0.0103 | 0.0000 | 0.0015 | 0.0000 |
| October | Cave | 0.0000 | 0.0000 | 0.0000 | 0.0000 | 0.0000 | 0.0000 |
| October | Falling Water | 0.0000 | 0.0000 | 0.0000 | 0.0000 | 0.0000 | 0.0000 |
| October | Sylamore | 0.0082 | 0.0007 | 0.0000 | 0.0000 | 0.0000 | 0.0000 |
| October | Tomahawk | 0.0000 | 0.0000 | 0.0000 | 0.0032 | 0.0000 | 0.0000 |
| October | Water | 0.0000 | 0.0000 | 0.0000 | 0.0000 | 0.0000 | 0.0000 |
